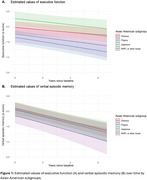# Differences in cognitive performance among diverse Asian Americans in Northern California

**DOI:** 10.1002/alz70857_098694

**Published:** 2025-12-24

**Authors:** Alexander Ivan B. Posis, Kristen M. George, Hilary L. Colbeth, Batool M. Rizvi, L. Paloma Rojas‐Saunero, Rifat B. Alam, Oanh L. Meyer, Paola Gilsanz, María M. M. Corrada, Rachel A. Whitmer

**Affiliations:** ^1^ University of California, Davis, Davis, CA, USA; ^2^ UCLA Fielding School of Public Health, University of California, Los Angeles, CA, USA; ^3^ University of California, Davis School of Medicine, Sacramento, CA, USA; ^4^ Kaiser Permanente Northern California Division of Research, Pleasanton, CA, USA; ^5^ University of California, Irvine, Irvine, CA, USA

## Abstract

**Background:**

Asian Americans comprise multiple ethnic groups with unique psychosocial and environmental exposures that likely result in differential rates of cognitive change. However, current research typically aggregates Asian Americans into a single group, which may inadvertently obscure potential cognitive differences. We compared cognitive performance across diverse Asian Americans subgroups in Northern California.

**Method:**

These analyses include 786 self‐identified Asian American participants of the harmonized the Kaiser Healthy Aging and Diverse Life Experiences and *LifeAfter90* studies (2018‐2022). Ethnicity was ascertained via self‐report. Executive function (EF) and verbal episodic memory (VM) were repeatedly measured using the Spanish and English Neuropsychological Scales and z‐scored to baseline. We used analysis of covariance tests to compare baseline cognitive function across subgroups, adjusting for pairwise comparisons. Linear mixed‐effects models for cognitive function were fit to assess subgroup differences over time. All models adjusted for age, gender and education. We calculated standardized mean differences (SMD) in cognitive function to quantify differences between ethnic groups.

**Result:**

Overall mean age was 80.5±9.4 years, 55% were women, and self‐reported ethnicities were 52% Chinese, 18% Filipino, 22% Japanese, and 8% Native Hawaiian, Pacific Islander or other Asian ethnic subgroup (NHPI, or other Asian). At baseline, there were significant differences between groups for EF (F‐statistic = 11.12; *p* < 0.01) and VM (F = 2.96; *p* =  0.03). The largest difference in EF was between Filipino and Japanese participants with Filipino participants showing significantly lower baseline EF scores (SMD = ‐0.85; 95% CI ‐1.08, ‐0.62). No significant differences for baseline VM were found after correction for pairwise comparisons (*p*'s > 0.18). Over an average follow‐up of 2.4±2.1 years, there were no group differences in cognitive change for EF (F = 1.11, *p* =  0.34) or VM (F = 0.15, *p* =  0.93; Figure 1).

**Conclusion:**

Results highlight disparities in baseline cognitive performance, particularly for baseline EF, that persisted over time among diverse Asian Americans. Disaggregated results are necessary to continue to understand and reduce disparities in cognitive function among Asian Americans.